# Association between sex hormones regulation‐related SNP rs12233719 and lung cancer risk among never‐smoking Chinese women

**DOI:** 10.1002/cam4.3772

**Published:** 2021-02-17

**Authors:** Ying Qian, Li Xie, Lei Li, Tienan Feng, Tengteng Zhu, Ruoyang Wang, Yuqing Yang, Baosen Zhou, Herbert Yu, Biyun Qian

**Affiliations:** ^1^ Hongqiao International Institute of Medicine Shanghai Tongren Hospital and School of Public Health Shanghai Jiao Tong University School of Medicine Shanghai China; ^2^ Clinical Research Institute Shanghai Jiao Tong University School of Medicine Shanghai China; ^3^ Department of Epidemiology China Medical University School of Public Health Shenyang China; ^4^ Cancer Epidemiology Program University of Hawaii Cancer Center Honolulu Hawaii USA; ^5^ Clinical Research Promotion and Development Center Shanghai Hospital Development Center Shanghai China

**Keywords:** Chinese women, lung cancer, never‐smoking, sex hormones regulation, SNP

## Abstract

**Background:**

The mechanism of rapidly increased non‐small cell lung cancer (NSCLC) among never‐smoking Chinese women has not been elucidated. Ovarian sex steroid hormones have been suggested to counteract lung cancer development, and sex hormone‐binding globulin (SHBG) is essential in sex hormones regulation. This study aims to exploring single nucleotide polymorphisms (SNPs) in genomic regions associated with SHBG concentrations that contributed to never‐smoking female NSCLC.

**Methods:**

Candidate genes were selected by a genome‐wide association (GWAS) meta‐analysis and gene expression profiles of never‐smoking NSCLC of Chinese women. The candidate SNPs limited to common minor allele frequency (MAF), missense variant, ethnic heterogeneous distribution, and SNPs were genotyped using the TaqMan method. A two‐stage case‐control design was adopted for exploration and validation of associations between candidate SNPs and risk of NSCLC. All participants were never‐smoking Chinese women. Chi‐square test and multivariate logistic regression were applied.

**Results:**

Beginning with 12 genomic regions associated with circulating SHBG concentrations and gene expression profiles from never‐smoking NSCLC in Chinese women, candidate SNP rs12233719 and rs7439366 both located in candidate gene UGT_2_B_7,_ which may be related to circulating SHBG concentrations and cancer risk, were identified. A two‐stage case‐control study was conducted in Shenyang and Tianjin represented as the training stage and validation stage, respectively. Under the dominant model, compared to individuals with the wild G/G genotype, the adjusted OR of those with the T allele was 1.58 (95% CI: 1.15–2.16) in Chinese Shenyang training set, and was 1.49 (95% CI: 1.02–2.18) in Chinese Tianjin validation set, both accompanied with a significant trend relationship consistently. UGT2B7 was upregulated in female NSCLC patients’ tumor tissues and was associated with a poor prognosis in NSCLC.

**Conclusion:**

Our findings indicated that a sex hormones regulation‐related SNP rs12233719 was associated with never‐smoking female lung cancer risk, which might partially explain NSCLC‐susceptibility in Chinese women.

## Graphical abstract

In this study, by conducting a two‐stage case‐control study, we found that a sex hormones regulation related SNP rs12233719 was associated with never‐smoking female lung cancer risk, which might partially explain NSCLC‐susceptibility in Chinese women.

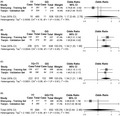

## 1 INTRODUCTION

Lung cancer is both the most common cancer and the leading cause of death from cancer in China and worldwide.^1^, ^2^ Research has shown genetic factors, lifestyle, and environment acting together in the non‐small cell lung cancer (NSCLC) development, including genetic variations and alterations, tobacco, air pollution, lung diseases history, nutrition, and occupational exposure to radon, asbestos, and radiation.^3^

The incidence of lung cancer in Chinese women with low smoking prevalence is unexplainable high.^4^ Previous study reported that compared to an older age at menopause, a younger age had an increased risk of lung cancer.^5^, ^6^ Based on another prospective epidemiologic study, among the never‐smoking female patients with lung adenocarcinoma, the premenopausal women had poor prognosis,^7^ postulating the beneficial effects of ovarian sex steroid hormones. Women have a better survival of lung cancer than men, and recently, both cancer‐specific survival and overall survival of late‐stage NSCLC in women was reported significantly improved by estrogen monotherapy therapy. A meta‐analysis including five prospective cohorts found a protective role of hormone therapy use in prognosis of lung cancer, and the pooled hazard ratio (HR) of the mortality rate was 0.80 (95% CI: 0.69–0.92).^8^ Accumulated evidence has suggested that a potential role of ovarian sex steroid hormones in female NSCLC.

Sex hormone‐binding globulin (SHBG) is a glycoprotein, having the capabilities to bind 17 beta‐hydroxysteroid hormones with high affinity, including testosterone and estradiol. And its concentration is able to regulate the bound‐hormone and free‐hormone states, playing as a transport carrier and regulating biological activities of sex hormones.^9^ Previously study has identified genetic variations of SHBG contributed to hormone‐sensitive cancer risk,^10^ such as prostate^11^ and breast.^12^ While the study about the impact of the genetic variation on SHBG concentrations and never‐smoking female NSCLC risk has not been evaluated before. We aimed to fill this gap by conducting a two‐stage case‐control study to explore never‐smoking female NSCLC‐susceptibility and 12 genomic regions associated with plasma SHBG concentrations, including 1p13.3, 2p16.3, 2p23.3, 4q13.2, 7q21.3, 8q21.13, 10q21.3, 12p21.1, 15q26.2, 17q21.32, 17p13.1, and Xq22.3, which were all identified by a GWAS meta‐analysis.^13^

## 2 METHODS

### 2.1 Study design

In this study, we adopted a two‐stage case‐control design^14^ in Chinese Shenyang and Tianjin, respectively. In stage 1, we aimed to obtain a crude measure of association between candidate SNPs and lung cancer risk. For stage 2, by conducting a propensity score matching, the association between SNP and lung cancer risk was validated in an independent validation population adjusted for other potential confounders.

### 2.2 Study subjects

In stage 1, Chinese Shenyang training stage, including 417 cases and 368 controls, was recruited from April 2011 to July 2015 in Shenyang, China. Inclusion criteria of cases were never‐smoking female who aged over 18 years, newly diagnosed NSCLC patients with histologically confirmation, without radiotherapy and chemotherapy. Inclusion criteria of controls were recruited from residents who lived in the same or nearby communities, healthy participants without cancer. For stage 2, Chinese Tianjin validation stage, was conducted at Tianjin Medical University Cancer Hospital, including 282 cases and 282 controls, as well as approved by ethical review. The recruitment of research participants was the same as mentioned above from January 2006 to May 2011. All subjects were genetically unrelated to the Han ethnic Chinese. Demographic characteristics were collected by structured questionnaires through an in‐person interview, and a peripheral blood sample (10 ml) was required for each participant collected by an ethylenediaminetetraacetic acid (EDTA) vacutainer tube. This study was approved by the Medical Ethics Committees of Human Studies at Tianjin Medical University Cancer Hospital, and written informed consent was signed by each participant.

### 2.3 RNA extraction and transcriptome sequencing

Eleven never‐smoking female NSCLC patients with paired tumor and adjacent tissues were used for RNA extraction and transcriptome sequencing. Differentially expressed were identified in the cuffdiff 2.2.1 based on the criterion of being significantly alteration (False discovery rate *p* < 0.05 and absolution of fold changes >1.5). Sequencing data were processed in general pipelines.^15^

### 2.4 SNP selection and genotyping

Based on the information of NCBI dbSNP and 1000 genomes project,^16^ SNPs were selected committed to the following criteria: (a) located in 12 genomic regions associated with plasma SHBG concentrations; (b) located in the genes differentially expressed in tumor and adjacent tissues in never‐smoking female NSCLC; (c) located in the genes with function of related to SHBG concentration as well as sex hormones regulation; (d) SNP function class limited to missense; (e) MAF>0.05 in CHB according to 1000 genomes project; (f) ethnic heterogeneous distribution between Chinese and Caucasian. The genotyping of the selected genetic polymorphisms was using TaqMan method reported previously.^17^

### 2.5 Bioinformatics analysis

Bioinformatics analysis was performed from publicly available data sets. The combined data from GSE32863 and GSE37764 after batch normalization was used to explore *UGT_2_B_7_* expression level in tumor and paired adjacent normal samples. GSE32863 was a gene expression profiling data set of paired samples from lung adenocarcinoma patients, including 16 paired samples of Asian never‐smoking women. GSE37764 contained a high‐throughput multidimensional sequencing study data of primary non‐small cell lung adenocarcinoma tumors and adjacent normal tissues of six never‐smoking Korean female patients. Meanwhile, GSE11969 and GSE13213 was obtained from the Gene Expression Omnibus (GEO) database which contained 17 and 7 never‐smoking female Japanese adenocarcinoma patients with EGFR mutant, respectively. The combined data from GSE11969 and GSE13213 after batch normalization was used to explore the relationship of *UGT_2_B_7_* expression and diagnosis of lung cancer.

### 2.6 Statistical analysis

Comparisons between cases and controls for continuous variables with a normal distribution (presented as the mean ± SD) were conducted by using the independent sample *t*‐test or Mann–Whitney *U* test, as appropriate. Propensity score matching (PSM) analysis was conducted by using exact match method, and standard mean difference was used to examine the balance of covariate distribution between cases and controls. Hardy–Weinberg equilibrium was calculated in each control data sample. Odds ratio (OR) and corresponding 95% confidence intervals (95% CI) were calculated to evaluate the associations between SNPs and NSCLC risk. To explore the independent effect of SNPs on the risk of NSCLC, we adjusted for potential confounding including age, family history of cancer by using multivariable conditional logistic regression model. HR was calculated to evaluate the associations between *UGT_2_B_7_* expression and diagnosis of never‐smoking female NSCLC. Statistically significant was defined as a two‐sided *p*‐value < 0.05. SAS version 8.2 ((SAS Institute Inc.), R software (version 4.0.2), and GraphPad Prism 6.0 (GraphPad Software Inc.) were used in the study analysis.

## 3 RESULTS

### 3.1 Selection of candidate genes

**FIGURE 1:**
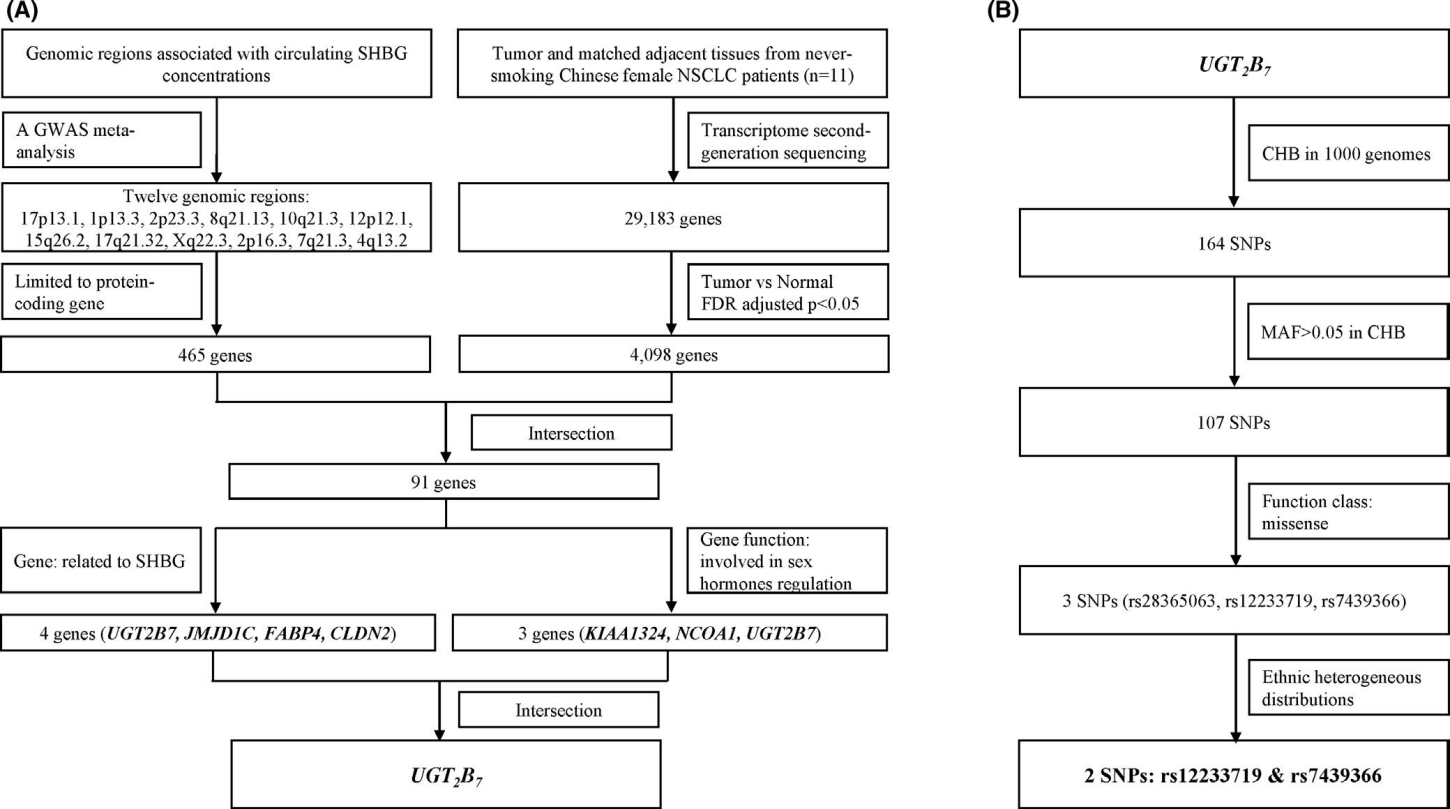
Study approach and selection of genes and SNPs

Figure 1 shows the diagram of how to select candidate genes. Beginning with 12 genomic regions associated with circulating SHBG concentrations, 465 genes were identified. Meanwhile, results from the transcriptome sequencing analysis from never‐smoking Chinese female NSCLC patients shown that *UGT_2_B_7_* mRNA was upregulated in tumor tissues (data were not shown), 4098 genes were identified. Finally, 91 candidate genes that may be related to circulating SHBG concentrations and cancer risk were selected. Considering the gene function, only *UGT_2_B_7_* was included, which was related to SHBG concentrations and sex hormones regulation by reported study (Figure 1).

### 3.2 Selection of candidate SNPs

**FIGURE 2:**
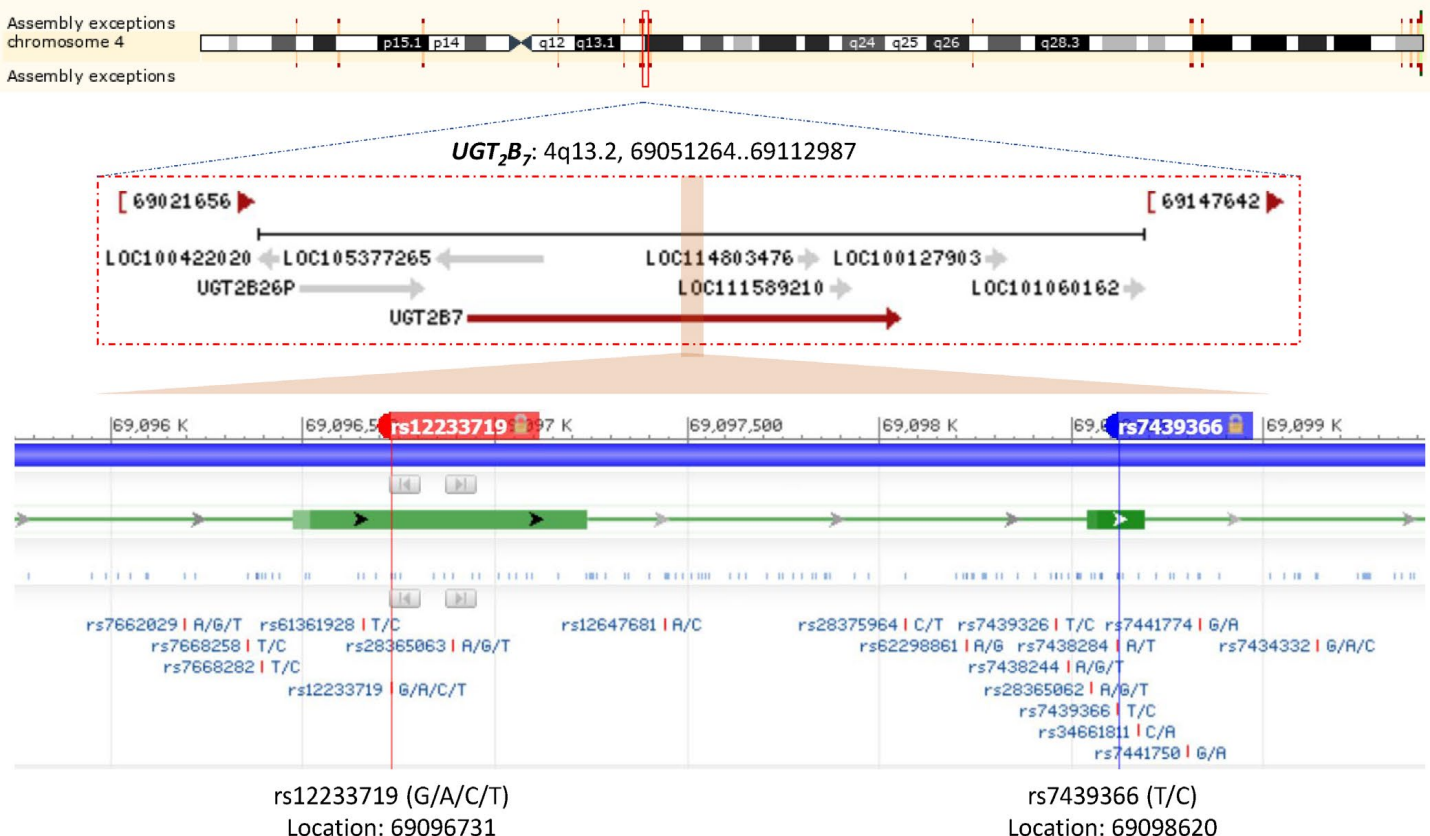
The location information of candidate gene and SNPs. UGT_2_B_7_ was located in 4q13.2, while SNPs rs12233719 and rs7439366 were annotated by red and blue tags, respectively

Beginning with candidate gene *UGT_2_B_7_*, combined with information from 1000 genomes, Figure 1B shows the diagram of how to select candidate SNPs. Finally, two SNPs, rs12233719 and rs7439366 both committed to common missense variants, were identified in our study (Figure 2). Hardy–Weinberg equilibrium test is shown in Table S1.

### 3.3 Association between rs12233719 and never‐smoking NSCLC risk in Chinese women

**FIGURE 3:**
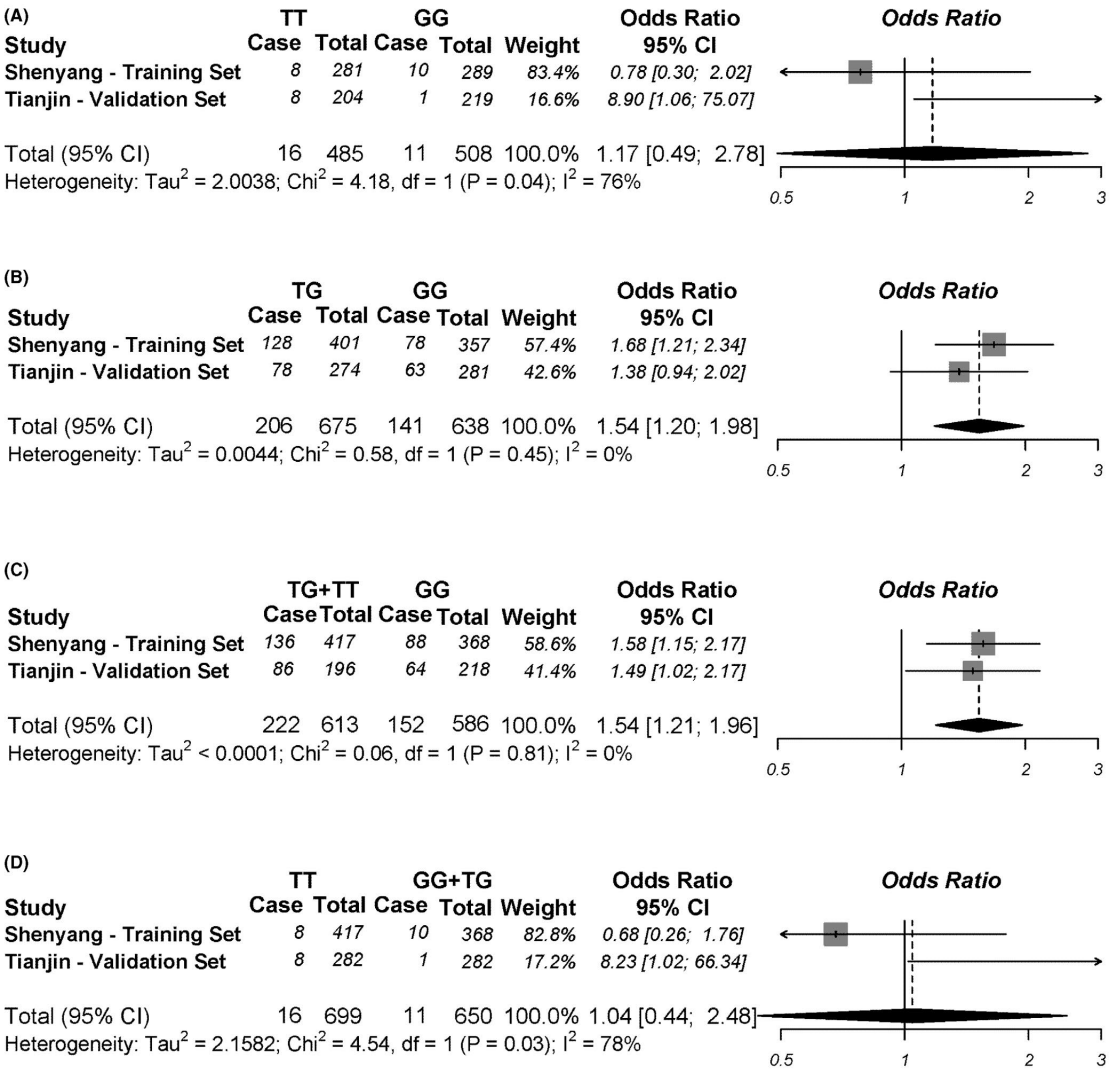
Forest plots for association between SNP rs12233719 G>T polymorphism and the risk of never‐smoking non‐small cell lung cancer in Chinese women in four different genetic models: (A) homozygous (TT vs. GG), (B) heterozygous (TG vs. GG), (C) dominant (TG + TT vs. GG), and (D) recessive (TT vs. GG + TG)

We conducted a two‐stage case‐control study in Chinese Shenyang (417 cases and 368 controls) and Tianjin (282 cases and 282 controls) as training and validation stage, respectively. Propensity score matching (PSM) analysis was conducted to balance the distribution of age in Tianjin validation set (Table S2). Under the dominant model, compared to individuals with the wild G/G genotype of rs12233719, the adjusted odds ratio (OR) of those with the T allele was 1.58 (95% CI: 1.15–2.16) in Chinese Shenyang training set, and was 1.49 (95% CI: 1.02–2.18) in Chinese Tianjin validation set. The trend analysis showed a significant relationship in both data sets (Table 1). In the meta‐analysis of combined training and validation set, the T allele of rs12233719 was associated with increased never‐smoking lung cancer risk in Chinese women, and the combined OR of the dominant model was 1.54 (95% CI: 1.21–1.96) (Figure 3). For rs7439366, no significant association was found (Table S3).

**TABLE 1:** Association between rs12233719 (G>T) and never‐smoking NSCLC risk in women of Chinese Shenyang training set and Chinese Tianjin validation set

Genotype	Case	Control	*p*‐value	OR (95% CI)	Adjusted OR (95% CI)^a^
***Chinese Shenyang training set***	**417**	**368**	**0.006**		
GG	273 (66.75)	279 (76.02)		Reference (1.00)	Reference (1.00)
TG	128 (31.30)	78 (21.25)		**1.68 (1.21–2.33)**	**1.68 (1.21–2.34)**
TT	8 (1.96)	10 (2.72)		0.82 (0.32–2.10)	0.78 (0.30–2.01)
***P for trend***			**0.021**		
**Dominant model**			**0.004**		
GG	273 (66.75)	279 (76.02)		Reference (1.00)	Reference (1.00)
TG+TT	136 (33.25)	88 (23.98)		**1.58 (1.15–2.17)**	**1.58 (1.15–2.16)**
**Recessive model**			0.478		
GG+TG	401 (98.04)	357 (97.28)		Reference (1.00)	Reference (1.00)
TT	8 (1.96)	10 (2.72)		0.71 (0.28–1.82)	0.68 (0.26–1.74)
***Chinese Tianjin validation set***	**282**	**282**	**0.016**		
GG	196 (69.48)	218 (77.30)		Reference (1.00)	Reference (1.00)
TG	78 (27.70)	63 (22.30)		1.38(0.94–2.02)	1.38(0.94–2.02)
TT	8 (2.80)	1 (0.40)		**8.87(1.10–71.51)**	**8.90(1.01–71.87)**
***P for trend***			**0.019**		
**Dominant model**			**0.036**		
GG	196 (69.48)	218 (77.30)		Reference (1.00)	Reference (1.00)
TG+TT	86 (30.52)	64 (22.70)		**1.49(1.02–2.18)**	**1.49(1.03–2.18)**
**Recessive model**			**0.019**		
GG+TG	274 (97.20)	281 (99.60)		Reference (1.00)	Reference (1.00)
TT	8 (2.80)	1 (0.04)		**8.18(1.02–65.8)**	**8.23(1.02–66.29)**

Abbreviations: CI, Confidence interval; OR, Odds ratio.

^a^ Adjusted by age, family history of cancer.

### 3.4 UGT_2_B_7_ mRNA expression in NSCLC

**FIGURE 4:**
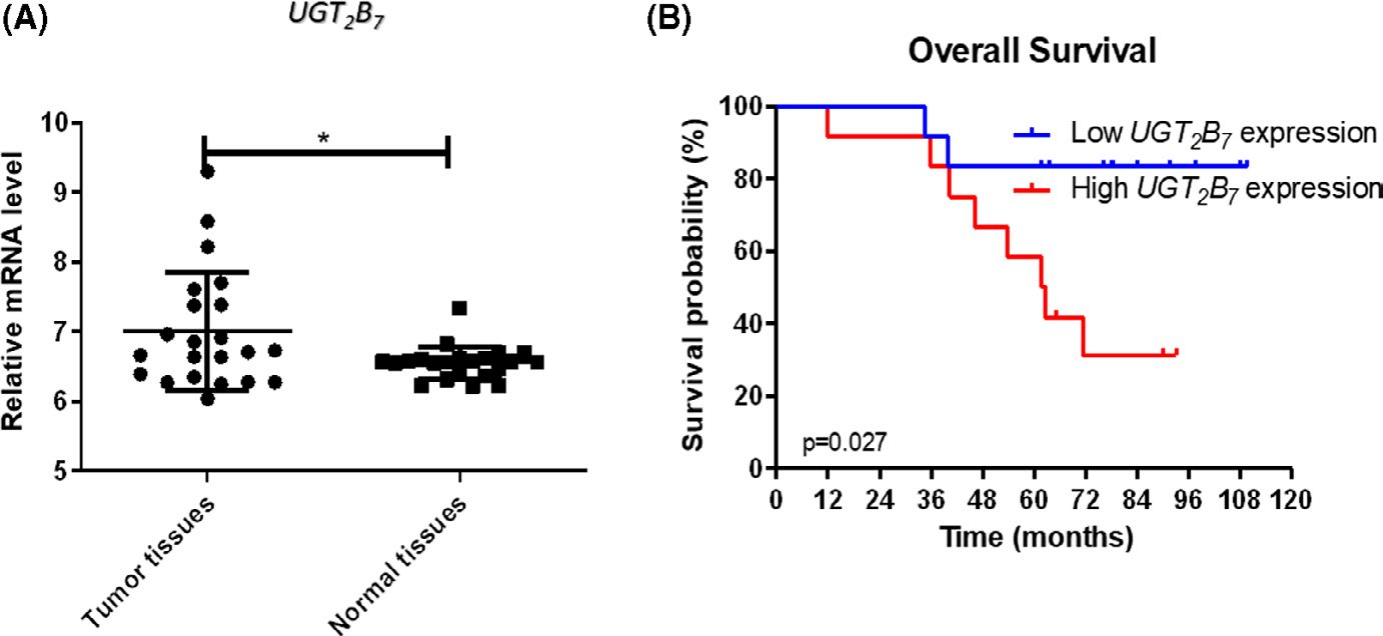
*UGT_2_B_7_* expression profile in NSCLC. A, Expression of *UGT_2_B_7_* in tumor and adjacent tissues (Tumor vs. normal tissues = 22 vs. 22); B, Association between *UGT_2_B_7_* expression overall survival (High vs. low expression = 24 vs. 24)

In the batch normalized combined data set of GSE32863 and GSE37764, compared to adjacent normal tissues, *UGT_2_B_7_* was high expressed in tumor tissues (*p* = 0.012) (Figure 4A). This result was consistent with our transcriptome sequencing analysis from 11 paired never‐smoking female NSCLC samples (data were not shown). Then, the relationship between *UGT_2_B_7_* expression and diagnosis was explored in combined data from GSE11969 and GSE13213 after batch normalization. Comparing to low *UGT_2_B_7_* expression, patients with high *UGT_2_B_7_* expression had significant poor overall survival among never‐smoking female Asian lung adenocarcinoma patients with EGFR mutation (HR = 4.80, 95% CI = 1.18–14.21, *p* = 0.027) (Figure 4B).

## 4 DISCUSSION

In this study, by conducting a two‐stage case‐control study, we explored SNPs in genomic regions that were associated with SHBG concentrations contributed to never‐smoking female NSCLC. Our findings indicated that sex hormones regulation‐related SNP rs12233719 was associated with NSCLC risk among never‐smoking Chinese women, which might partially explain NSCLC‐susceptibility in Chinese women.

In this study, rs12233719 and rs7439366 both located in *UGT_2_B_7_* at locus 4q13.2 were identified as the candidate gene and SNPs. The *UGT_2_B_7_* gene is located on chromosome 4q13.2 with the length of 16 kb, and is widely distributed in the breast, lung, kidney, and intestine, which is considered to be highly polymorphic gene.^18^ *UGT_2_B_7_* consists of six exons and five introns and can code for 529 amino acid residues. *UGT_2_B_7_* was a well‐known pharmacogene belonging to the uridine diphosphate glucuronyltransferase (UGT) gene family,^19^ which played an essential role in estrogen regulation, and its enzymatical activity was found changed by genetic variants.^20^ Notably, *UGT_2_B_7_* had unique specificity for estrogens and catechol estrogens, including estradiol, estriol, 4‐OH‐estrone, and 4‐OH‐estradiol, serving a significant role in the elimination of exogenous and endogenous estrogens.^21^ While genetic variation in *UGT_2_B_7_* was recently reported to increase SHBG levels in premenopausal women with oral contraceptive use.^22^ Many studies have revealed associations between genetic variation in *UGT_2_B_7_* and cancer risk, including breast and prostate cancer. Thus, factors affecting the expression and enzyme activity of *UGT_2_B_7_* may play an important role in tumorigenesis, of which the *UGT_2_B_7_* mutation may be one of the essential factors involved in the occurrence and development of cancer.

Our study first identified SNP rs12233719 G>T polymorphism being a risk factor of never‐smoking female NSCLC, which could partially explain the NSCLC‐susceptibility to never‐smokers in Chinese women. In the coding region of the *UGT_2_B_7_*, rs12233719 was another missense mutation at position 211 (G211T). A G>T transversion at position G211T was associated with an amino acid change from Ala71 to Ser71, which resulted in a change from a lipophilic residue to a hydrophilic residue.^19^, ^23^ However, differ from previous studies that reported the not any statistically significant association between *UGT_2_B_7_* rs12233719 SNP in all genetic models and breast cancer risk,^24^ our study found that SNP rs12233719 G>T polymorphism being a risk factor of never‐smoking female NSCLC. A possible explanation might be that rs12233719 is a non‐synonymous SNP and substitutes amino acid 71 from alanine to serine and alters the physical and chemical properties of this position,^25^ suggesting that polymorphism probably affect the expression and enzymatic activity of *UGT_2_B_7_*. While the mechanism of rs12233719 impact on NSCLC was unknown, more functional experiments and further studies are urgent to elucidate the underlying linkage. Besides, according to the source of biological samples and the population of SNPs selection, the findings of our study were limited to Chinese female population, which should be interpreted cautiously.

We found rs7439366 was not associated with never‐smoking female NSCLC risk in our study. Polymorphism of rs7439366 is a common missense located in exon 2 of *UGT_2_B_7_*, which may rise the enzymes with either histidine (H) or tyrosine (Y) at amino acid 268.^26^ Rs7439366 with a T to C transversion at nucleotide 802, according to Tyr to His conversion at residue 268. The C alleles were 0.489 and 0.732 in Caucasians and Japanese, respectively.^27^ Many previous studies evaluated the role of *UGT_2_B_7_* rs7439366 SNP on cancer risk. To explore the role of *UGT_2_B_7_* SNPs in cancer susceptibility, a meta‐analysis study pooled all eligible studies and found no significant association was observed in all genetic models. Further subgroup analysis found that rs7439366 was associated with colorectal cancer risk.^28^

Consideration should also be given to potential limitations in this study. First, we failed to perform vivo or vitro experiment to confirm the relationship between rs12233719 and SHBG concentrations. Second, we only adjusted a few potential confounders in the multivariable logistics regression model, which may lead to bias in an unpredictable direction. Potential residual confounders within the individual participants may affect the estimate, such as menopause status. Menopausal status is closely correlated with women's lung cancer risk, but we were not able to assess this because of the limited data source. Thus, larger studies, especially prospective studies, are warranted in the future. Third, we failed prospectively estimated the sample size for two‐stage case‐control study, as well as the transcriptome sequencing experiment. Due to the retrospective characteristics of case‐control study design, we conducted the exploration and validation in a two‐stage case‐control study for screening SNPs without prospective sample size estimation. Meanwhile, the transcriptome sequencing in 11 paired NSCLC patients and controls samples for screening differential genes in nonsmoking female NSCLC was limited several years ago due to limited clinical samples. Besides, it was a preliminary exploration, as well as lack of proper reference data for sample size estimation. Therefore, the results should be interpreted with caution. In the next step, we will try to expand the sample size based on this study.

## 5 CONCLUSIONS

In summary, we found that rs12233179, involved in sex hormones regulation, was associated with NSCLC risk among never‐smoking Chinese women. Our finding suggests that the SNP rs12233179 in sex hormones regulation may be significant in Chinese female NSCLC, and provides new insight into the possible role of ovarian sex steroid hormones in lung cancer.

## 6 ETHICS APPROVAL AND CONSENT TO PARTICIPATE

This study was approved by the Medical Ethics Committees of Human Studies at China Medical University, and written information consent was signed by each participant. All participants provided written informed consent.

## AUTHOR CONTRIBUTIONS

Conceptualization, YQ, LX, and BQ; Data curation, YQ, LX, and BQ; Formal analysis, YQ, LX, LL, and BQ; Methodology, YQ, LX, LL, TZ, TF, and BQ; Project administration, TZ and BQ; Supervision, HY and BQ; Writing—original draft, YQ, LX, HY, and BQ. The final manuscript was revised by all authors, and this version was approved to be published.

## Supporting information

Table S1‐S3

## Data Availability

The data sets used and analyzed are available by request from the corresponding author in this study.
