# Supplementary material for: Association between sex hormones regulation‐related SNP rs12233719 and lung cancer risk among never‐smoking Chinese women
Source: Cancer Med. 2021 Feb 17;10(5):1880–8. doi: 10.1002/cam4.3772 (PMC7940208; doi:10.1002/cam4.3772)
Supplement: Supplementary file 1 — Table S1‐S3 [file CAM4-10-1880-s001.docx]

**Table S1. Hardy-Weinberg equilibrium analysis in each dataset**

| **SNPs** | **Dataset** | **Genotype in controls** | | ***P-value*** |
| --- | --- | --- | --- | --- |
| Rs12233719 | *Chinese-Shenyang training set* |  |  | 0.119 |
|  |  | GG | 279 (76.02) |  |
|  |  | GT | 78 (21.25) |  |
|  |  | TT | 10 (2.72) |  |
|  | *Chinese-Tianjin validation set* |  |  | 0.629 |
|  |  | GG | 218 (77.30) |  |
|  |  | GT | 63 (22.30) |  |
|  |  | TT | 1 (0.40) |  |
| Rs7439366 | *Chinese-Shenyang training set* |  |  | 0.277 |
|  |  | TT | 40 (11.17) |  |
|  |  | TC | 173 (48.32) |  |
|  |  | CC | 145 (40.50) |  |
|  | *Chinese-Tianjin validation set* |  |  | 0.381 |
|  |  | TT | 30 (10.60) |  |
|  |  | TC | 144 (51.10) |  |
|  |  | CC | 108 (38.30) |  |

**Table S2. Age distributions in training and validation cohort**

| **Dataset** | **Age (Mean±std)** | | ***P-value*** |
| --- | --- | --- | --- |
|  | **Case** | **Control** |  |
| *Chinese-Shenyang training set* | 56.78±11.46 | 54.31±12.18 | 0.004 |
| *Chinese-Tianjin validation set (before PSM)* | 60.50±9.24 | 47.80±16.00 | <0.001 |
| *Chinese-Tianjin validation set*  *(after PSM)* | 59.35±9.31 | 59.34±9.30 | 1.000 |

**Table S3. Association between rs7439366 (T>C) and never-smoking lung cancer risk in women of Chinese-Shenyang training set**

| **Genotype** | **N (%)** | | ***P-value**** | ***OR (95% CI)*** | ***Adjusted OR (95% CI)***** |
| --- | --- | --- | --- | --- | --- |
|  | **Case (N=417)** | **Control (N=368)** |  |  |  |
| ***UGT_2_B_7_* rs7439366 (T>C)** | |  | 0.280 |  |  |
| TT | 47 (12.11) | 40 (11.17) |  | Reference (1.00) | Reference (1.00) |
| TC | 165 (42.53) | 173 (48.32) |  | 0.81 (0.51-1.30) | 0.80 (0.50-1.29) |
| CC | 176 (45.36) | 145 (40.50) |  | 1.03 (0.64-1.66) | 1.01 (0.63-1.63) |
| ***P for trend*** |  |  | 0.425 |  |  |
| **Dominant model** |  |  | 0.689 |  |  |
| TT | 47 (12.11) | 40 (11.17) |  | Reference (1.00) | Reference (1.00) |
| TC+CC | 341 (87.89) | 318 (88.83) |  | 0.91 (0.58-1.43) | 0.90 (0.57-1.41) |
| **Recessive model** |  |  | 0.181 |  |  |
| TT+TC | 212 (54.64) | 213 (59.50) |  | Reference (1.00) | Reference (1.00) |
| CC | 176 (45.36) | 145 (40.50) |  | 1.22 (0.91-1.63) | 1.21 (0.90-1.61) |

* Two-side, and calculated by χ^2^ test; **Adjusted by age; OR=Odds ratio; CI=Confidence interval.
